# Structural, Functional, and Cellular Analysis of a Case of Acute Zonal Occult Outer Retinopathy (AZOOR)

**DOI:** 10.3390/biomedicines13071521

**Published:** 2025-06-21

**Authors:** Mattia Iuliano, Marco Lombardo, Benedetto Falsini, Jacopo Sebastiani, Michele D’Ambrosio, Francesco Martelli, Andrea Cusumano

**Affiliations:** 1 Ophthalmology Unit, Department of Experimental Medicine, University of Rome Tor Vergata, 00133 Rome, Italy; 2 Macula & Genoma Foundation USA, New York, NY 10017, USA; 3 Macula & Genoma Foundation, 00196 Rome, Italy; 4 Department of Cardiovascular and Endocrine-Metabolic Diseases and Ageing, National Institute of Health, 00161 Rome, Italy

**Keywords:** adaptive optics transscleral flood illumination, AO-TFI, AZOOR, retinal pigment epithelium, photoreceptors, OCT, OCTA, dark-without-pressure

## Abstract

**Background:** Adaptive optics transscleral flood illumination (AO-TFI) enables in vivo, non-invasive, high-resolution imaging of retinal pigment epithelium (RPE) and photoreceptor (PR) cells, paving the way for a new potential characterization of retinal diseases. This study aimed to analyze RPE and PR cells in a case of acute zonal occult outer retinopathy (AZOOR) using AO-TFI. **Methods:** A patient affected by AZOOR underwent a comprehensive eye examination, perimetry, electroretinography (ERG), autofluorescence, and optical coherence tomography (OCT) during the acute phase (T0). After three years (T1), OCT angiography (OCTA) and AO-TFI were also performed. Voronoi analysis was utilized to quantify RPE and PR cells. **Results:** At T0, OCT revealed interruptions in the ellipsoid zone (EZ) of the right eye, while the structure of the left eye appeared normal. Perimetry and ERG were abnormal in both eyes. At T1, OCT indicated recovery of the EZ in the right eye, while thinning of the ONL persisted. Perimetry and mfERG values remained below normative limits. OCTA exhibited globally reduced vessel density in the inner retina of the right eye. AO-TFI demonstrated reduced PR density in affected areas despite preserved EZ, while RPE cell density appeared unaffected. **Conclusion:** AO-TFI enabled a detailed visualization and quantification of macular RPE and PR cells, providing valuable insights into the pathophysiology of AZOOR.

## 1. Introduction

Acute zonal occult outer retinopathy (AZOOR) is a retinal disorder with an indeterminate etiology, characterized by the rapid and significant loss of one or more extensive zones in the outer retinal layers. This condition typically presents in a sectoral pattern and reveals minimal observable changes during a fundoscopic examination [1]. AZOOR predominantly affects the peripapillary region that surrounds the optic nerve, with approximately one-third of patients experiencing bilateral involvement, often accompanied by notable asymmetry. In the initial stages, patients may exhibit only subtle retinal alterations; however, visual field defects can deteriorate over time, while central visual acuity generally remains preserved. Some individuals may remain asymptomatic despite considerable dysfunction in specific areas of the outer retina. The condition commonly manifests with an acute or subacute onset of photopsia and visual field abnormalities [2]. Gass first delineated this condition through a study involving thirteen patients, predominantly young women, who reported a sudden onset of photopsia and scotomas [3].

The concept of AZOOR was further elaborated by including it in the AZOOR complex, which encompasses several related disorders, including multiple evanescent white dot syndrome, punctate inner choroidopathy, idiopathic multifocal choroiditis, acute macular neuroretinopathy, and acute idiopathic blind spot enlargement syndrome. In contrast to AZOOR, lesions associated with other conditions in the AZOOR complex are visible and correlate with observable changes in the fundus, usually indicating disorders of the retinal pigment epithelium (RPE) and photoreceptors (PRs) [4].

A longitudinal study indicated that seventy-eight percent of the eyes achieved stability in visual symptoms within six months of presentation. Overall, the prognosis for AZOOR appeared favorable, with sixty-eight percent of patients maintaining a visual acuity of 20/40 or better in the affected eye [5]. As the condition advances, individuals with AZOOR may reveal fundoscopic changes, including visible chorioretinal atrophy, typically well-demarcated, often accompanied by diffuse intraretinal pigment migration [6]. Currently, there is no established treatment protocol for AZOOR, and the necessity or efficacy of any therapeutic intervention remains indistinct. Treatment options include corticosteroids, nonsteroidal immunomodulatory therapies, and antiviral medications, among others.

The underlying cause of AZOOR remains enigmatic, and the patterns of visual loss and retinal involvement do not consistently correlate, even with any definitive vascular issues. One report suggests a potential association between AZOOR and polycythemia [7]. The increased prevalence of AZOOR in young women, who are generally more prone to autoimmune disorders, combined with a relatively high prevalence of concurrent autoimmune diseases, indicates a possible autoimmune component to the condition. However, the diverse responses of patients to corticosteroids and immunomodulatory therapies could imply that additional non-autoimmune factors may also contribute to the disorder.

Various structural and functional techniques can help in assessing AZOOR. These examinations include automated perimetry, optical coherence tomography (OCT), OCT angiography (OCTA), fundus autofluorescence (FAF), fluorescein angiography (FA), indocyanine green angiography (ICGA), electroretinography (ERG), and adaptive optics (AO) [8,9].

AO retinal imaging is notably improving our understanding of the retina in vivo. This advanced technology achieves optical resolutions of two micrometers or less, enabling detailed measurements of cellular and sub-cellular characteristics related to the structure and function of the retina. Previously, such insights were primarily confined to post-mortem tissue analysis or studies conducted on non-human models. Moreover, AO retinal imaging serves as a powerful tool for investigating disease mechanisms and identifying potential biomarkers, potentially providing more sensitive outcome measures for clinical interventions. In vivo observation of the human retina at the cellular level could be crucial for the early detection and effective treatment of retinal diseases, such as AZOOR [10].

Despite significant advancements in AO systems, clinical imaging of various retinal cell types continued to face challenges, primarily due to the low signal-to-noise ratio (SNR) associated with transpupillary illumination. Imaging individual cells encountered considerable obstacles, including ocular aberrations that diminish lateral resolution, artifacts caused by eye motion, and the inherent low contrast of transparent cells. Additionally, much of the light entering the pupil is either absorbed or reflected at the boundary of the PR segments [11]. AO with transscleral flood illumination (AO-TFI) offers a solution by providing cellular-resolution, label-free, high-contrast images of retinal layers while avoiding the limitations linked to prolonged exposure times. This method uses transscleral flooding to illuminate the retina, significantly improving the SNR of various retinal structures compared to traditional transpupillary illumination techniques. The light that penetrates the sclera creates an oblique illumination of the retina, captured using a trans-pupillary AO full-field camera system. This represents a novel microscopy concept and an innovative instrument for rapid, in vivo, non-invasive imaging of the RPE with high contrast and cellular resolution [12]. Quantitative analysis may utilize a Voronoi diagram, a tessellation pattern that divides a plane into distinct cells based on proximity to specific points. Therefore, AO-TFI can potentially enhance our understanding of retinal diseases involving PR and RPE and establish new morphological endpoints for diagnosis and therapy.

In this report, we examine a case of AZOOR through functional and structural assessments, including AO-TFI.

## 2. Materials and Methods

A 31-year-old female patient diagnosed with AZOOR presented with an acute onset of central scotoma in her right eye following episodes of cold and headache. She underwent a comprehensive eye examination during the acute phase (T0) and again three years later (T1). At T0, the examinations included assessing the best corrected visual acuity (BCVA) using standard ETDRS charts, performing a slit lamp examination of both the anterior and posterior segments, conducting a 30-2 frequency doubling technology (FDT) perimetry (Humphrey Matrix, Carl Zeiss Meditec, Jena, Germany), multifocal electroretinography (mfERG) with Retimax (CSO, Florence, Italy), fundus autofluorescence (FAF), spectral-domain optical coherence tomography (OCT), fluorescein angiography (FA), indocyanine green angiography (ICGA) using Spectralis (Heidelberg Engineering, Heidelberg, Germany), and ultra-widefield (UWF) retinography with Clarus 500 (Carl Zeiss Meditec, Jena, Germany). At T1, additional exams were conducted, including swept-source UWF-OCT and OCT-angiography (OCTA) using the VG200S (SVision Imaging, Luoyang, Henan, China) and AO-TFI with Cellularis Discovery (EarlySight SA, Geneva, Switzerland). Voronoi analysis was employed to quantify the density of RPE and PR cells across all macular sectors at an eccentricity of 5 degrees (central, superior, temporal, inferior, nasal).

A paired *t*-test was conducted to evaluate the overall differences in PR and RPE densities between the right and left eyes across all macular zones. Statistical analysis was performed on the combined dataset. Due to the limited sample size, individual comparisons for specific macular zones were not made. The significance level for statistical testing was set at *p* < 0.05. All statistical analyses were performed using GraphPad Prism (version 9.5.1; GraphPad Software, San Diego, CA, USA). The Ethics Committee of the National Institute of Health, Rome, Italy, granted ethical approval for the use of AO-TFI imaging in human subjects (authorization 2023/0059828 from the National Ethics Committee for clinical trials of public research bodies).

## 3. Results

At T0 and T1, the patient’s BCVA remained stable at 0.0 logMAR in both eyes. The anterior segment examination was normal, and the fundus examination demonstrated no noticeable changes. The 30-2 FDT perimetry indicated a globally reduced differential light sensitivity with enlargement of the blind spot in the right eye. The mfERG revealed decreased amplitude, which affected the right eye, particularly in the nasal sector. The damage observed in these exams persisted at the last follow-up, indicating no substantial improvement.

**Figure 1:**
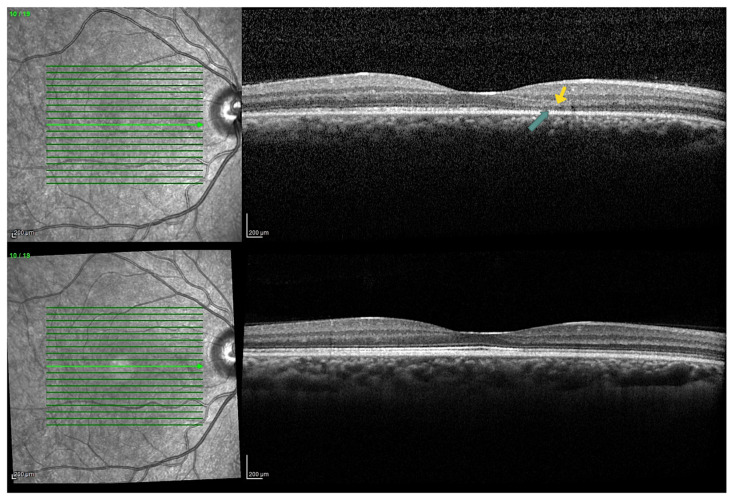
The macular OCT scan at T0 (**top**) reveals a disruption in the parafoveal ellipsoid zone (EZ) and a reduction in the thickness of the outer nuclear layer (ONL). The yellow arrow in the top image indicates the area of ONL thinning, while the green arrow highlights the disruption of the EZ. In the follow-up OCT at T1 (**bottom**), signs of restoration are observed in the EZ; however, the thickness of the ONL remains partially reduced.

The OCT scan of the right eye showed a diffuse interruption of the ellipsoid zone (EZ) with associated thinning of the outer nuclear layer (ONL), mainly in the nasal parafoveal region. No changes were observed in the left eye. FAF, FA, and ICGA results were normal in both eyes. The patient tested positive for anti-alpha-enolase antibodies. The visual symptoms and OCT findings demonstrated gradual spontaneous improvement over three months. Three years after the initial assessment, OCT imaging revealed a restoration of the ellipsoid zone interruption in the right eye. However, the thinning of the ONL persisted (Figure 1). Additionally, the inner retinal thickness was reduced compared to the healthy left eye.

**Figure 2:**
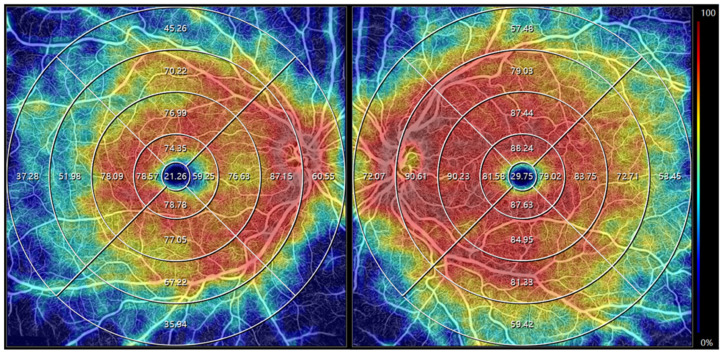
OCTA indicates a decreased inner retinal vessel density in the right eye compared to the left. On the color scale, red signifies areas of higher vascular density, while blue denotes regions of lower vessel density. The adjacent color scale specifically illustrates vascular density levels.

OCTA demonstrated globally reduced vessel density in the inner retina (superficial and deep capillary plexuses) of the right eye, particularly pronounced in the nasal parafovea compared to the left eye (Figure 2).

**Figure 3:**
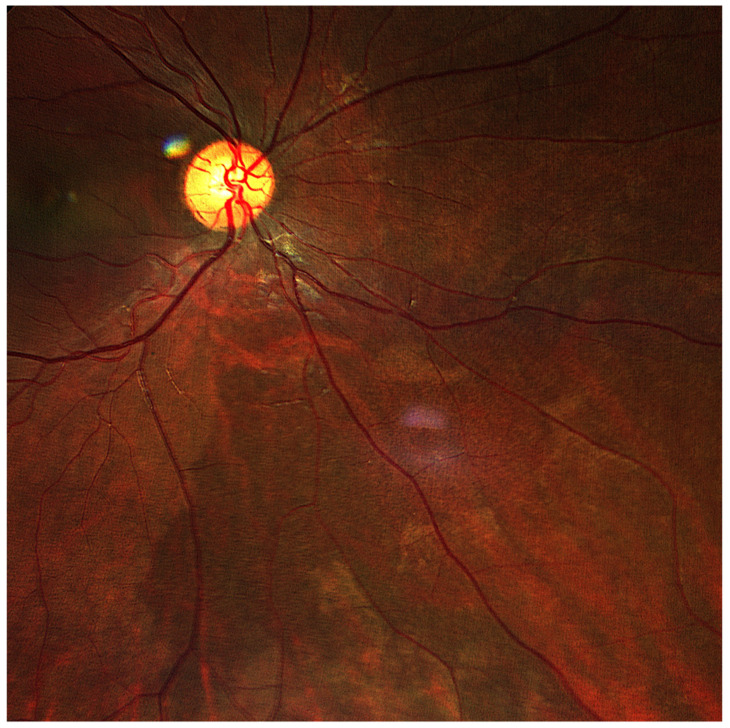
Retinography shows a DWP fundus lesion in the inferior-nasal mid-periphery of the right eye.

Ultra-widefield (UWF) retinography revealed a dark-without-pressure (DWP) spot in the nasal mid-periphery (Figure 3).

**Figure 4:**
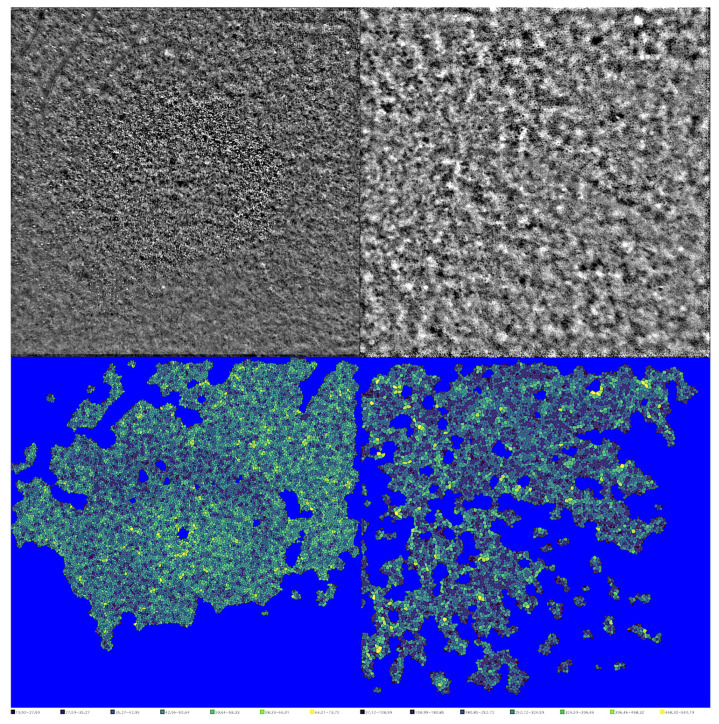
AO-TFI images (**top**) of PR (**top left**) and RPE (**top right**) in the macular area. Below are the relative segmentations obtained through Voronoi analysis (**bottom left** and **bottom right**). At the bottom of the image, the cellular density scale is displayed.

Ultra-widefield OCT showed an attenuation of the ellipsoid zone in this area. Perimetry and mfERG values remained below normative limits. The patient underwent AO-TFI to assess macular PR and RPE in both eyes (Figure 4).

Quantitative analysis of the images using the Voronoi diagram indicated a reduction in PR density in the affected eye compared to the contralateral healthy eye (*p* < 0.05). No statistically significant difference was found in the RPE evaluation between the two eyes (*p* > 0.05) (Table 1).

## 4. Discussion

Although AZOOR was first described in 1992, its causes and treatment remain under investigation. It is not a homogeneous condition; it comprises various variants that exhibit different behaviors. Certain variants progress aggressively, resulting in decreased visual acuity, an increased risk of recurrence, a relative afferent pupillary defect, and, in rare instances, the development of a neovascular membrane [13,14,15]. Conversely, other variants may resolve independently [16]. The response to corticosteroid treatment in AZOOR is not always predictable [17,18]. This unpredictability could be associated not only with the timing of the treatment but also with the varying underlying etiologies of the condition [19,20]. Differential diagnoses such as acute macular neuroretinopathy (AMN) and autoimmune retinopathy (AIR) were considered. However, the observed pattern of abnormalities, including the ERG findings, visual field defects, and fundoscopic features, did not align with either AMN or AIR. In cases of AMN, structural changes are typically confined to the outer plexiform and outer nuclear layers and are often associated with characteristic parafoveal lesions, which were not seen in this case [21,22]. Similarly, AIR usually involves more diffuse retinal dysfunction and displays distinct ERG patterns [23]. Consequently, the combination of clinical, functional, and imaging findings supported a diagnosis of AZOOR and effectively ruled out these alternative conditions.

In cases of AZOOR, a multimodal imaging analysis is essential due to the complexity of the condition and the need for differential diagnosis [3]. OCT typically reveals abnormalities in PRs, including disruptions in the EZ [8]. Visual field loss is a key characteristic of AZOOR, making it strongly recommended to conduct a visual field test to confirm the diagnosis. Various types of defects may be present, with enlargement of the blind spot being the most frequently reported [9]. ERG is crucial for diagnosing AZOOR, as it often shows electrophysiological abnormalities in the affected eyes [6]. In some instances, there may be no visible changes to the retina during a fundus examination [3]. The literature has reported alterations in choriocapillaris perfusion following PR damage, and OCT angiography has proven to be a valuable tool in the diagnostic evaluation of AZOOR [1]. Previous studies indicated that the main changes observed in AZOOR suggest a dysfunction and degeneration of the outer segments of photoreceptors [24,25]. Anti-retinal antibodies have been detected in approximately 42% of patients with AZOOR [26]. Our patient tested positive for anti-alpha-enolase antibodies and reported experiencing viral prodromes before the onset of the ophthalmic manifestations of AZOOR. These findings suggest a potential autoimmune etiology, which is considered one of the most likely causes of AZOOR [2,26]. The detection of autoantibodies in serum involved an immunological test, specifically a Western blot. This process begins with electrophoresis on a denaturing polyacrylamide gel using 1 microgram of each target recombinant protein. The proteins are separated based on their molecular weight and then transferred onto a membrane. After incubation with the serum sample, the membrane is washed to eliminate any nonspecifically bound autoantibodies. It is then incubated with a secondary anti-human antibody that is conjugated to horseradish peroxidase. The antibodies captured on the membrane are visualized via chemiluminescent reaction following substrate incubation [27]. The patient also presented a dark-without-pressure (DWP) fundus lesion, which has been reported in patients with autoimmune diseases [28]. The presence of this lesion, primarily involving the EZ, further supports the use of AO-TFI in this study. In OCTA, we observed reduced superficial and deep vessel density in the nasal parafoveal sector [29]. This area corresponded to a decrease in ONL thickness. A possible explanation may stem from the self-regulation mechanism of retinal flow due to the reduction in the inner retina thickness. This change could, in turn, result from the remodeling of the inner retina following damage to the PRs. Since AZOOR is primarily a non-vascular disease, the impairment of vessel density caused by the autoregulation of blood flow following the inner retina remodeling could explain this finding in chronic cases of AZOOR.

Using conventional diagnostic retinal imaging, a significant discrepancy was observed between the structural findings and the functional measurements obtained through perimetry and mfERG, indicating a persistent visual impairment despite the apparent improvement in the right eye. An abnormal ERG is a crucial diagnostic criterion for AZOOR. It is important to note that mfERG can sometimes detect early functional changes before clinical symptoms arise [30]. Generally, diminished mfERG responses are observed in regions associated with visual field defects. In contrast, full-field ERG usually exhibits lower sensitivity compared to mfERG and might seem normal, especially when visual field defects are slight [6].

Regarding perimetry, AZOOR is linked to a variety of visual field loss patterns, which mainly result in either an expansion of the blind spot or are associated with it. Additionally, it is essential to emphasize that the damage detected in mfERG and visual field evaluations generally does not improve over time in patients diagnosed with AZOOR [31]. In some cases, damage may gradually progress, and the chorioretinal atrophy can lead to severe visual field loss [32].

During the OCT assessment, the patient demonstrated only a persistent thinning of the ONL with minimal rarefaction of the EZ, which posed challenges for quantification. AO has the potential to significantly aid in assessing damage at the PR level in AZOOR, and the Voronoi test facilitated accurate measurement of the extent of the damage [33]. AO-TFI utilizes specialized optical techniques to achieve distinct lateral resolution in visualizing retinal cells. It facilitated detailed observation of the RPE and PRs. By generating a dark-field image, this method significantly minimized interference from the neurosensory retina, offering a clearer view of these important cell layers. In fact, the transscleral illumination bypasses the Stiles–Crawford effect, enabling visualization of the RPE that conventional AO devices with transpupillary illumination cannot achieve [12].

AO-TFI revealed a reduced PR density in previously affected areas, which could explain the remaining functional impairment. No statistically significant differences were observed in the RPE at AO-TFI, supporting the theory that PRs are the primarily involved site. Not all reported patients with AZOOR exhibit damage to PRs when assessed using AO, and a resolution in AO without PR damage appears to be correlated with improvements in functional exams, including mfERG and visual field tests [33]. The longitudinal evaluation from T0 to T1 highlighted the importance of conducting early, comprehensive baseline assessments to interpret long-term outcomes accurately. Without an initial detailed assessment of structural and functional characteristics, subtle residual deficits may be mistakenly viewed as primary abnormalities instead of consequences of the disease. This emphasizes the essential role of coordinated imaging and functional testing, not only for monitoring progress but also for gaining a better understanding of the underlying pathophysiology. The findings from this single case report, although limited, may offer valuable insights relevant to a broader population of patients with AZOOR. It is well established that in cases of AZOOR, functional deficits can persist even when conventional imaging shows apparent anatomical restoration [5,8,14,24]. This observation aligns with our findings. While a previous AO study noted a case of AZOOR exhibiting both anatomical and functional recovery, such outcomes are relatively rare [33].

In contrast, the case we present illustrates what may be a more typical presentation of AZOOR, marked by ongoing functional impairment and residual structural damage that can only be detected at the cellular level through AO-TFI. This highlights the potential of high-resolution imaging to uncover subtle yet clinically significant pathologies that might otherwise be missed. Additionally, it supports the use of AO-TFI for more comprehensive diagnostic and prognostic evaluations in AZOOR patients.

There may be a relationship between the cellular damage observed at the level of PRs by AO-TFI and the extent of residual functional damage. Further monitoring studies using AO-TFI may be useful to assess whether any progression of damage detected in functional tests for some forms of AZOOR is linked to a corresponding progression of cellular damage over time. The differences observed with AO-TFI may yield insights into the various manifestations of the disease. In some instances, there may be residual damage to the PRs, while in others, the condition may resolve completely.

Specific forms of AZOOR can also cause significant damage to the RPE, which may persist even after the acute condition resolves. Employing AO-TFI can enhance our capacity to assess the diverse prognoses and variations of this condition more effectively. Therefore, it is essential to comprehend the potential correlation between persistent structural damage in AO-TFI and the risk of recurrence, which could facilitate adequate follow-up for the patients. Currently, there is no established treatment protocol for AZOOR, as the underlying cause remains uncertain. Some studies have reported variable responses to treatments such as corticosteroids, antiviral agents like acyclovir, and immunomodulatory drugs such as adalimumab [17,18,34,35]. However, the effectiveness of these therapies is still debated and lacks consistent evidence. This uncertainty underscores the potential value of AO imaging techniques, particularly AO-TFI, in future clinical management. AO-TFI could be essential for monitoring structural changes at the cellular level, thereby enhancing our understanding of treatment effects and guiding therapeutic decisions for AZOOR. Performing AO-TFI imaging during both the acute phase and the immediate post-acute stages could provide valuable insights into early structural changes, particularly involving the RPE. This method would allow us to assess whether any initial damage to the RPE was temporary and later resolved spontaneously. Longitudinal imaging in this manner could improve our understanding of AZOOR’s natural progression and support more accurate prognostic evaluations by capturing early, potentially reversible cellular changes that might not be detectable at later stages. This study has several limitations. As a case report, it is not possible to generalize the findings related to AO-TFI for all individuals diagnosed with AZOOR who exhibit residual damage in functional exams. Moreover, despite its numerous diagnostic and therapeutic advantages, AO has not yet become a standard diagnostic tool in routine clinical practice. Additionally, this technique has not achieved widespread standardization.

## 5. Conclusions

Multimodal imaging is essential for a comprehensive understanding of residual damage and retinal reorganization in AZOOR. AO-TFI allows for a non-invasive, in vivo, detailed observation and quantification of RPE and PRs. This innovative technique may reveal subclinical cellular variations that are intricately linked to functional changes, thereby aiding in the characterization and deeper understanding of the underlying causes while also tracking the progression of AZOOR. In our specific case, conventional imaging revealed a notable difference between structure and function, indicating that the functional impairment appeared more evident than the observable anatomical damage. However, AO-TFI indicates more substantial remaining structural damage, potentially accounting for the functional alterations seen in the patient. Utilizing AO-TFI to analyze this category of patients during the acute phases of the disease may greatly enhance our overall understanding of AZOOR complex mechanisms. Furthermore, analyzing a broader cohort of AZOOR patients at a cellular level could also facilitate the identification of disease subtypes, which is essential for improving early diagnosis and clinical outcomes.

## Figures and Tables

**Table 1:** Density of PR and RPE expressed in regions/mm^2^ across all macular sectors calculated using Voronoi analysis. The values presented correspond to the single measurements in the corresponding macular zone. The *p*-values indicate a significant difference between eyes for PR, but not for RPE.

Macular Zone	Right Eye	Left Eye	*p*-Value	Right Eye	Left Eye	*p*-Value
	PR Density (Regions/mm^2^)			RPE Density (Regions/mm^2^)		
Central	9913	10,410	0.013	3296	3990	0.148
Superior	9644	10,344		4062	4066	
Temporal	9648	10,290		3840	4048	
Inferior	9497	10,324		3953	3934	
Nasal	9009	10,660		4024	4286	

## Data Availability

Data available on request due to restrictions (privacy reasons): the data presented in this study are available on request from the corresponding author.
